# Elementary, Atomic-Level Friction Processes in Systems with Metallic Inclusions—Systematic Simulations for a Wide Range of Local Pressures

**DOI:** 10.3390/ma14164351

**Published:** 2021-08-04

**Authors:** Małgorzata Gzik-Szumiata, Tadeusz Szumiata, Dmitrij Morozow, Roman Szewczyk

**Affiliations:** 1 Department of Physics, Faculty of Mechanical Engineering, University of Technology and Humanities in Radom, Stasieckiego 54, 26-600 Radom, Poland; m.gzik@uthrad.pl; 2 Department of Machine Technology, Faculty of Mechanical Engineering, University of Technology and Humanities in Radom, Stasieckiego 54, 26-600 Radom, Poland; d.morozow@uthrad.pl; 3 Institute of Metrology and Biomedical Engineering, Faculty of Mechatronics, Warsaw University of Technology, A. Boboli 8, 02-525 Warsaw, Poland; roman.szewczyk@pw.edu.pl

**Keywords:** atomic-level friction, metallic inclusions, Lennard–Jones potential, simulations

## Abstract

In this work, simulations of friction at the atomic level were performed to evaluate the influence of inclusions coming from metallic nanoadditives in the friction pair. The simple 2D model was applied considering appropriate values of Lennard–Jones potential parameters for given sets of interacting atoms. The real sliding pairs were replaced by effective equivalents consisting of several atoms. The calculations were based on the pseudo-static approximation. The simplicity of the model enabled to repeat the fast calculations in a very wide range of local pressures and for several types of atomic tribopairs. The performed simulations demonstrated a strong dependence of the coefficient of friction (COF) on the atomic environment of the atoms constituting a tribopair. It was confirmed theoretically that the Mo-Fe pair is characterized by lower atomic COF than Fe-Fe, Cu-Fe, and Ag-Fe pairs. This points to the great applicational potential of metallic molybdenum coating applications in tribological systems. Moreover, it was demonstrated that, although Cu-Cu and Ag-Ag pairs are characterized by relatively high COF, they lower the friction as inclusions in Fe surfaces.

## 1. Introduction

In the last quarter of a century, a renaissance of the interest in experimental studies on friction (especially in nanoscale) has been observed [1,2,3,4]. In parallel, great progress in atomic-level simulations of frictional processes has been made. In order to describe the elementary interactions between atoms in friction pairs, molecular dynamics (MD) analysis is usually utilized with great success [1,5,6,7,8,9,10,11,12]. An especially interesting task is to predict the impact of metallic additives in reducing the value of the coefficient of dry sliding friction as well as wear intensity. In [13], it was unequivocally proven that the addition of Cu nanoparticles can reduce, at the atomic level, the effective value of friction coefficient and wear of iron–iron tribopair—especially in the case of low-speed dry sliding. The applied MD simulations demonstrated the formation of a sliding layer after the disintegration of Cu nanoparticles. A very recent paper (2020, [14]) also reports a positive impact of atoms from Cu nanoparticles on the frictional properties of an Fe-Fe sliding system at elevated temperatures and under high loads as well as the profiting tendency of lowering the local temperature during friction. These MD simulations were based on the method of quantum semi-empirical embedded atom (EAM) potentials [15,16].

Although MD simulations are at present a standard modern tool for the modelling of frictional phenomena at the atomic level, semiclassical simple models with a limited number of degrees of freedom are still in use [17,18,19,20,21,22,23]. The most important of them are those based on the dynamics of harmonic oscillators, i.e., the 1D and 2D Prandl–Tomlinson models (also called the “independent oscillators model”), the Frenkel–Kontorova model (atomic chain in periodic potential), thermally activated Prandtl–Tomlinson model extensions, the combined Frenkel–Kontorova–Tomlinson model [24], as well as 2D models based on limited-size matrices of atoms [25]. These models are especially suitable for the description of the friction force microscopy (FFM) principle and the interpretation of the atomic friction results obtained using FFM. They also allow to discuss and analyze (sometimes even analytically) such crucial questions like physical reasons of the deviations from the Leonardo da Vinci–Guillaume Amontons law of dry (Coulomb) friction. Another kind of simple model is based on a rigid-body description of nanoparticles [26] with commensurate and incommensurate atomic interfaces, as well as models related to continuum medium mechanics [27,28]. The significance of simplified tribological models is appreciated when one needs to repeat the calculations for many values of different parameters (e.g., of load force). In such a case, large-scale MD simulations could be too time-consuming. Moreover, not only are MD simulations themselves demanding, but also the obtained numerical results are sometimes not easy to use for analysis and comparison. The main reasons are the fast jumps of frictional and load forces in the picosecond time domain and within the picometers range of sliding distance changes [11,13,29,30,31,32]. These discontinuities and lack of periodicity are a real challenge for the proper numerical treatment and comparison of the results for various tribopairs. They could be a source of errors in averaging lateral and normal forces and in determining the effective coefficient of friction (COF).

The main goal of the present work was to previse theoretically the frictional characteristics of several types of atomic tribopairs in a very wide range of load pressures. A scientific significance of the applied model is its time-efficiency, simplicity, and consideration of different atomic inclusions, which can occur after the disintegration of metallic nano-particles, very commonly used as modifiers of friction. We considered frictional pairs of Fe, Cu, Ag, and Mo atoms possessing different atomic surroundings in the first coordination zone in their crystalline structure. In general, the metals used as additives in the form of the fine particles (especially Cu and Ag) do not reveal themselves to have lower COF than iron or steel (as typical tribopairs in mechanical devices). Nevertheless, the softness of these metals and nanometric size of the particles enable the self-repair of rubbing surfaces, i.e., smoothing out the roughness at an atomic level. The geometry of the considered model strictly corresponds to such a situation. The motivation for the modelling within a simple 2D several atoms system was to avoid time-consuming MD simulations. Such an option allowed to consider more types of atomic tribopairs of metals and to obtain the results for a wider range of local stresses. Owing to the simplicity of the model, the main expectation was not to reproduce the absolute COF values, but to determine the relative tendencies when varying the kind of metallic atoms’ inclusions. The calculations were based on the fast pseudo-static algorithm, based on the series of equilibrium states being determined for each position of the slider. This approach was successfully employed in the case of the modelling of spin-dependent frictional phenomena [33].

## 2. Theoretical Model

**Figure 1:**
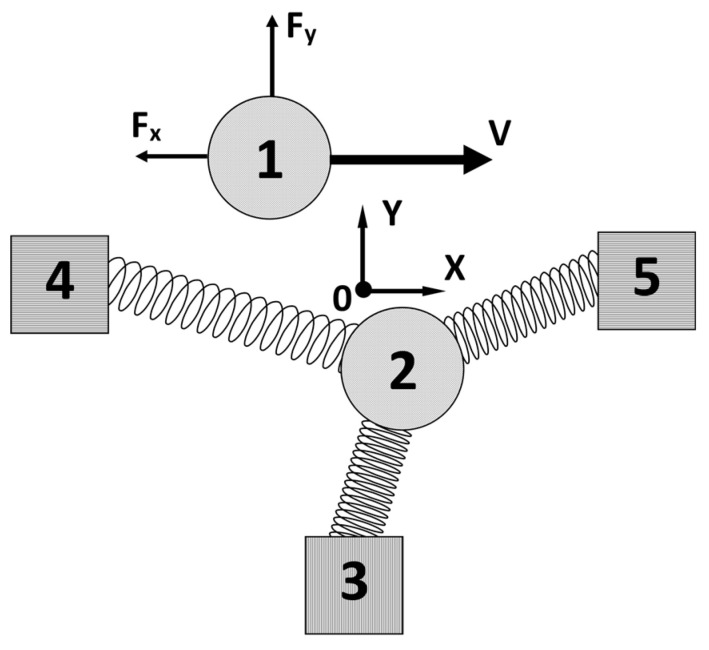
Atoms’ geometry of frictional pair in the pseudo-static 2D model: atom (1) of the slider and atom (2) of base elastically bonded to three rigid atoms (3,4,5).

In the frame of the simple model, the elementary friction at the atomic level can be illustrated by the scheme shown in Figure 1. Atom 1 represents a “slider” moving quasi-statically with a small, horizontal velocity *v* at a certain height over the massive “slab”. This atom interacts with atom 2 belonging to the “slab”. Atom 2 is elastically bounded to three neighbouring atoms (3,4,5) in 2D geometry. For simplicity, these atoms are assumed to be fixed—i.e., rigidly connected to the other part of the “slab”.

In order to describe the interatomic interactions, the standard Lennard–Jones (L–J) potential $U_{LJ}$ was used, which is also a standard choice for MD simulations [11,29] (nevertheless, some MD works are based on pairwise Morse potential [7,8]). In the case of metallic systems, the most appropriate description of interatomic interactions is realized by potentials within the embedded atom model (EAM) [15,16]. Nevertheless, such an approach for binary alloys engages seven functions: three pairwise interactions, two embedding functions, and two electron cloud contribution functions. Furthermore, parameters are necessary to consider angular-dependent, triatomic interactions originating from the geometry of quantum orbitals within the tight-bonding approach. That is why, because of the simplicity of the presented 2D tribological model, the Lennard–Jones potential was chosen. It requires a very limited number of parameters and can approximately describe both non-bonding and bonding states. The L–J potential energy is given by the following formula:

(1) $$U_{LJ}{\left(r_{ij}\right)}_{\varepsilon_{ij},\sigma_{ij}}=4\varepsilon_{ij}\left[{\left(\frac{\sigma_{ij}}{r_{ij}}\right)}^{12}-{\left(\frac{\sigma_{ij}}{r_{ij}}\right)}^{6}\right]$$

where $r_{ij}$ is a relative distance between atom i and j, $\varepsilon_{ij}$ is “bonding energy” (as a depth of the potential minimum), and $\sqrt[{6}]{2}\sigma_{ij}$ is the “length of the bond” (corresponding to the minimum of the potential). The effective values of both parameters have to be chosen for the “bonding case” (not for description of long-distance van der Waals interactions). When the pair (i,j) is heteroatomic, the approximate equations for binary Lorentz–Berthelot mixing rules [33,34,35] can be utilized:

(2) $$\varepsilon_{ij}=\sqrt{\varepsilon_{ii}\cdot \varepsilon_{jj}},\;\;\;\;\;\;\;\;\sigma_{ij}=\frac{\sigma_{ii}+\sigma_{jj}}{2},$$

where $\varepsilon_{ii},\;\varepsilon_{jj},\sigma_{ii},\;\mathrm{and}\;\sigma_{jj}$ are Lennard–Jones potential parameters for the homoatomic pairs.

In the case of 2D geometry, $U_{LJ}$ takes a form of the explicit function of the relative coordinates $\left(x_{ij},y_{ij}\right)$:

(3) $$U_{LJ}{\left(x_{ij},y_{ij}\right)}_{\varepsilon_{ij},\sigma_{ij}}=4\varepsilon_{ij}\left[\frac{\sigma_{ij}^{12}}{{\left(x_{ij}^{2}+y_{ij}^{2}\right)}^{6}}-\frac{\sigma_{ij}^{6}}{{\left(x_{ij}^{2}+y_{ij}^{2}\right)}^{3}}\right].$$

Thus, $F_{x}$ and $F_{y}$ components of the force acting on the atom i from the atom j can be expressed as follows:

(4) $$F_{x}{\left(x_{ij},y_{ij}\right)}_{\varepsilon_{ij},\sigma_{ij}}=-\frac{d}{dx_{ij}}U_{LJ}{\left(x_{ij},y_{ij}\right)}_{\varepsilon_{ij},\sigma_{ij}}=4\varepsilon_{ij}x_{ij}\left[\frac{12\sigma_{ij}^{12}}{{\left(x_{ij}^{2}+y_{ij}^{2}\right)}^{7}}-\frac{6\sigma_{ij}^{6}}{{\left(x_{ij}^{2}+y_{ij}^{2}\right)}^{4}}\right],$$

(5) $$F_{y}{\left(x_{ij},y_{ij}\right)}_{\varepsilon_{ij},\sigma_{ij}}=-\frac{d}{dy_{ij}}U_{LJ}{\left(x_{ij},y_{ij}\right)}_{\varepsilon_{ij},\sigma_{ij}}=4\varepsilon_{ij}y_{ij}\left[\frac{12\sigma_{ij}^{12}}{{\left(x_{ij}^{2}+y_{ij}^{2}\right)}^{7}}-\frac{6\sigma_{ij}^{6}}{{\left(x_{ij}^{2}+y_{ij}^{2}\right)}^{4}}\right].$$

To find the actual position of atom (2) of the “slab” changed via interaction with “sliding” atom (1), the quasi-static equilibrium conditions demand the vanishing of x and y components of the resultant force exerted on atom (2) by atoms (1), (3), (4), and (5):

(6) $$\begin{array}{c} F_{x}{\left(x_{2}-x_{1},y_{2}-y_{1}\right)}_{\varepsilon_{12},\sigma_{12}}+F_{x}{\left(x_{2}-x_{3},y_{2}-y_{3}\right)}_{\varepsilon_{23},\sigma_{23}}+\\ \;\;\;+F_{x}{\left(x_{2}-x_{4},y_{2}-y_{4}\right)}_{\varepsilon_{24},\sigma_{24}}+F_{x}{\left(x_{2}-x_{5},y_{2}-y_{5}\right)}_{\varepsilon_{25},\sigma_{25}}=0, \end{array}$$

(7) $$\begin{array}{c} F_{y}{\left(x_{2}-x_{1},y_{2}-y_{1}\right)}_{\varepsilon_{12},\sigma_{12}}+F_{y}{\left(x_{2}-x_{3},y_{2}-y_{3}\right)}_{\varepsilon_{23},\sigma_{23}}+\\ \;\;\;+F_{y}{\left(x_{2}-x_{4},y_{2}-y_{4}\right)}_{\varepsilon_{24},\sigma_{24}}+F_{y}{\left(x_{2}-x_{5},y_{2}-y_{5}\right)}_{\varepsilon_{25},\sigma_{25}}=0 \end{array}$$

This system of nonlinear algebraic equations was solved numerically with the Levenberg–Marquardt method using Mathcad software. As an output of this routine, the tables $X_{2}\left(x_{1}\right),\;Y_{2}\left(x_{1}\right)$ of “slab” atom (2) coordinates were obtained as a function of the “slider” atom (1) position. Inserting these values into Equations (4) and (5), one can obtain the tables $F_{x}$ and $F_{y}$ containing the values of x and y components of the force acting on the “slider” atom (1) from the “slab” atom (2):

(8) $$F_{x}\equiv F_{x}{\left(x_{1}-X_{2}\left(x_{1}\right),y_{1}-Y_{2}\left(x_{1}\right)\right)}_{\varepsilon_{12},\sigma_{12}},$$

(9) $$F_{y}\equiv F_{y}{\left(x_{1}-X_{2}\left(x_{1}\right),y_{1}-Y_{2}\left(x_{1}\right)\right)}_{\varepsilon_{12},\sigma_{12}}.$$

It has been assumed that, during the whole sliding process, the vertical position (“height”) of the atom is unchanged ($y_{1}=h$). For simplicity, the origin of the coordinates system was chosen as an equilibrium position of atom (2) in the unperturbed state. The initial distances between atom i=2 and atoms j=3, 4, 5 are described by corresponding “lattice constants” $a_{ij}=\sqrt[{6}]{2}\sigma_{ij}$.

**Figure 2:**
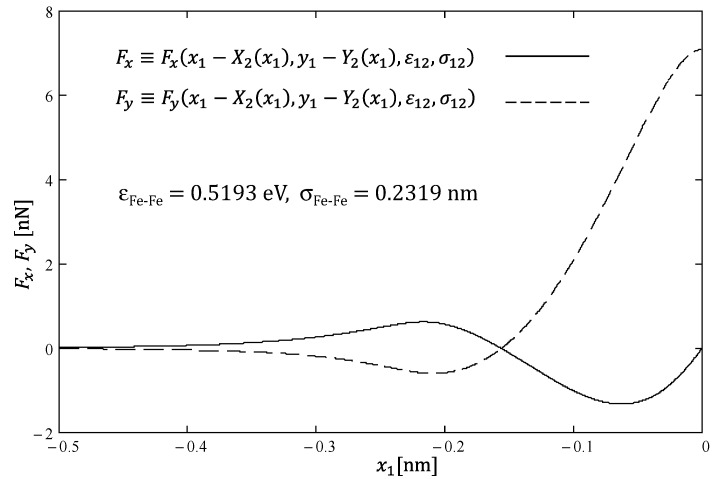
The exemplary dependences of lateral (*x*) and normal (*y*) forces acting on “sliding” atom (1) from the “slab” atom (2) in 2D iron “crystalline lattice” for *y*_1_ = *h* = 0.208 nm as a function of the position of atom (1).

The example of $F_{x}\left(x_{1}\right)$ and $F_{y}\left(x_{1}\right)$ curves in the case of all iron atoms and the height of “sliding” atom *y*_1_ = *h* = 0.208 nm is presented in Figure 2. When atom (1) is far to the left from atom (2) located at the “zero” point, the lateral force (*x*) acting on atom (1) is positive (rightwards), whereas the normal force is negative (downwards). This is a simple consequence of the attracting nature of (L–J) interatomic interactions for long distances. For shorter distances, the interactions become repulsive and much stronger (Figure 2). In this region, one deals with the real friction (negative leftwards *x*-force component) and the reaction to real pressing (positive upwards *y*-force component) acting on “sliding” atom (1). Simultaneously, the same mutual interaction is responsible for shifting atom (2) rightwards and downwards.

To calculate the average value of friction force $\langle F_{x}\rangle$ and load force $\langle F_{y}\rangle$, the following formulae were used:

(10) $$\langle F_{x}\rangle =\frac{1}{\frac{a_{23}+a_{25}}{2}}\cdot \int_{x_{01}}^{0}\mathrm{Minus}\left[-F_{x}{\left(X_{2}\left(x_{1}\right)-x_{1},Y_{2}\left(x_{1}\right)-y_{1}\right)}_{\varepsilon_{12},\sigma_{12}}\right]dx_{1},$$

(11) $$\langle F_{y}\rangle =\frac{1}{\frac{a_{23}+a_{25}}{2}}\cdot \int_{x_{01}}^{0}\mathrm{Plus}\left[-F_{y}{\left(X_{2}\left(x_{1}\right)-x_{1},Y_{2}\left(x_{1}\right)-y_{1}\right)}_{\varepsilon_{12},\sigma_{12}}\right]dx_{1},$$

where

$$\mathrm{Plus}\left(F_{y}\right)=\{\begin{array}{c} F_{y},\;\;F_{y}\geq 0\\ 0,\;\;F_{y}<0 \end{array}\;,\;\;\;\;\;\;\;\;\;\;\;\;\;\;\;\;\;\;\;\;\;\;\;\;\mathrm{Minus}\left(F_{x}\right)=\{\begin{array}{c} -F_{x},\;\;F_{x}\leq 0\\ 0,\;\;F_{x}>0 \end{array}.$$

Such an averaging procedure considers only the regions with real friction and load force. The lower limit of integral was taken as $x_{01}=-2a_{12}$—well to the left from the expected point of the sign change of the interaction force components. The choice of the upper limit at 0 point is a natural consequence of the quasi-static approach. In this case, the solution of the static Equations (6) and (7) for $x_{1}>0$ generate unphysical solutions, the form of which is close to the antisymmetric function. This would lead to the almost total vanishing of the mean friction force given by Equation (10) when integrating over the symmetrical region around the zero point. The sign change of the lateral force and *x*-coordinate of atom (2) (after passing the vicinity of “critical” zero point) corresponds to a very fast relaxation of the accumulated elastic energy in a real, dynamic friction process. This means that the region from zero point to, say, the half distance point from the next atom of the “slab” is an interval of virtual nonequilibrium states related to the processes of energy dissipation via the creation of phonons in the crystalline lattice. Such a rapid jump of the lateral force was predicted by the historical—but still useful—“spring model” proposed by the Prandtl and Tomlinson model [17,18,19]. This phenomenon, typical for atomic-level friction, was confirmed experimentally using force friction microscopy (FFM) [17], and the obtained spatial dependencies of the lateral force were of saw-tooth-like shape. The complex question about the nature of the atomic-scale “stick and slip” effect was also a matter of MD simulations [12]. The simple averaging procedure described by Equations (10) and (11) within the indicated limits of integration can be justified under the reasonable assumption that most of the accumulated elastic energy is rapidly transferred into the crystalline lattice after jump at $x_{1}=0$. The systematic MD study of this “stick-slip” behaviour was performed, e.g., in [34], including the asymmetry of the ascending and descending part of the friction force peak.

**Figure 3:**
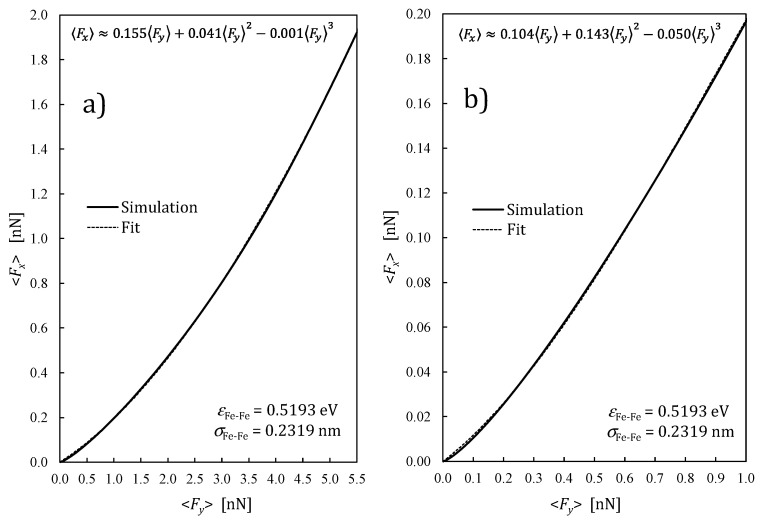
The simulated and polynomially fitted dependence of the averaged lateral (friction) force on the normal (loading) force in the case of iron–iron friction at the atomic level: (**a**) a wide range of load and (**b**) a low load range.

By changing the height *y*_1_ = *h* of “sliding” atom (1) over the “slab”, the series of simulations were performed for different ranges of the average load forces. The lowest force corresponded to the biggest height $h=a_{\mathrm{Fe}-\mathrm{Fe}}\approx \;$0.260 nm, and the highest force to $h=0.6\cdot a_{\mathrm{Fe}-\mathrm{Fe}}\approx \;$0.156 nm. The exemplary dependences of the averaged lateral friction force $\langle F_{x}\rangle$ on the averaged normal load force $\langle F_{y}\rangle$ are presented in Figure 3a,b for the case of iron–iron atomic friction. The results of simulations do not fulfil the commonly known Leonardo da Vinci–Amontons law (usually valid for macroscopic dry friction, called Coulomb friction):

(12) $$\langle F_{x}\rangle =\mu \langle F_{y}\rangle ,$$

where μ is the friction coefficient. In Figure 3a,b, it is clearly visible that the dependencies are not linear, whereas the nonlinear terms’ contribution is higher in the case of a lower load range (b) than for higher loads (a).

The observed nonlinearity leads to the non-constant coefficient friction:

(13) $$\mu \equiv \frac{\langle F_{x}\rangle }{\langle F_{y}\rangle }.$$

One can consider that, for the practical analysis, this coefficient is a function of a local load pressure calculated as follows:

(14) $$p=\frac{\langle F_{y}\rangle }{{\left(\frac{a_{23}+a_{25}}{2}\right)}^{2}},$$

where the denominator represents an elementary surface under the assumption that, at the surface, the atoms are ordered in a simple cubic lattice. This approach makes it easier to relate the results of the 2D model simulation to the real 3D friction data. Formula (14) considers possible differences in interatomic distances $a_{23}$ and $a_{25}$ (heteroatomic case) and requires the assumption that the geometry of atoms is the same in the x and z (i.e., third) direction.

When the load is being changed not from zero, but around a definite value, it would be more precise to introduce the differential atomic friction coefficient:

(15) $$\delta \mu =\frac{\delta \langle F_{x}\rangle }{\delta \langle F_{y}\rangle },$$

which, of course, is a function of load pressure.

## 3. Outcomes of Simulations and Discussion

**Figure 4:**
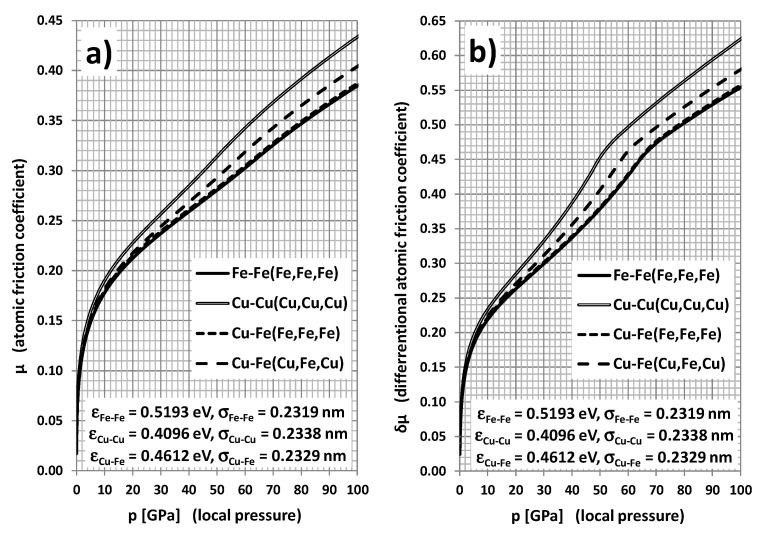
Regular (**a**) and differential (**b**) atomic friction coefficient dependence on local pressure for different atomic pairs: Fe-Fe(Fe,Fe,Fe), Cu-Cu(Cu,Cu,Cu), Cu-Fe(Fe,Fe,Fe), and Cu-Fe(Cu,Fe,Cu). Atoms specified in parentheses constitute a rigid environment of an “elastic” atom of the base.

In Figure 4, the pressure dependences of atomic friction coefficients (regular and differential) are displayed for the following pairs of atoms: Fe-Fe(Fe,Fe,Fe), Cu-Cu(Cu,Cu,Cu), Cu-Fe(Fe,Fe,Fe), and Cu-Fe(Cu,Fe,Cu), where the first atom represents “slider” (1); the second one is a “flexible” atom (2) of the “slab”; and in parentheses are the atoms (4), (3), and (5) of the “slab”. The dependence for pure iron–iron friction is a “reference curve” for all other cases. A very fast increase of atomic friction coefficient value is visible in the lower range of load pressure; however, for higher pressures as well, the value of the coefficient still grows. 

The load pressure scale in Figure 4 (and further figures) seems to be unexpectedly large at first glance. The maximal value (100 GPa) overcomes about 200 times the ultimate tensile strength (*UTS* = 0.54 GPa) and numerically is of the order of Young’s modulus value (*E* = 210 GPa) and shear modulus (*G* = 53 GPa) [36]. However, the simulated averaged pressure (by hanging the height h of the “slider” atom from the “slab”) is not an exerted macroscopic pressure, but a very local “atomic” pressure. If we consider the nanoroughness of the surfaces participating in friction, the effective contact surface can be, e.g., 1000 times smaller than a regular macroscopic surface of the plates. This explains the large increase of the local pressure and the destructive consequences of the friction. 

The empirical values of L–J parameters for Fe-Fe, Cu-Cu, Ag-Ag, and Mo-Mo atomic pairs were taken from the available literature [37,38,39,40,41,42,43,44,45]. Nevertheless, there is a rather significant spread of the values of “bonding energy” and “bonding length” L–J parameters reported in the literature. This is a consequence of different methods of estimation, e.g., from molecular dynamics simulations of the melting process, or commonly accessible data on cohesive energy, crystalline structure, and lattice constants.

All curves in Figure 4 (and in the next figures) demonstrate significant derogation from the Leonardo da Vinci–Amontons law of friction, which previews the constant friction coefficient. This simple rule refers to the macroscopic friction, whereas the present outcomes describe the friction at the atomic level. Nevertheless, it is not very hard to indicate qualitatively a transition between these two cases [46]. Although, according to Figure 4, the atomic friction coefficient increases with pressure, in reality, it would be accompanied by the increase of the contact surface owing to the strong deformations of surface asperities. This would lead to the stabilizing of the local pressure value and near-constant “saturation” value of effective macroscopic friction coefficient, following Amontons law. The visible increase of the friction coefficient in Figure 4 is a simple consequence of the nonlinearity of the friction force dependence on the loading force presented in Figure 3a,b. A very similar increase of atomic-scale COF was reported in [34] as a result of MD simulations for diamond surfaces.

The simulated curve for Cu-Cu atomic pair friction lays noticeably beyond the Fe-Fe curve. The higher values of the atomic friction coefficient (especially for higher pressures) seem to be a natural consequence of the fact that, in comparison with iron, the cooper is a softer metal (*E* = 117 GPa, *G* = 45 GPa, *UTS* = 0.22 GPa). Moreover, copper has a significantly lower melting temperature (*T*_Cu_ = 1085 °C) than iron (*T*_Fe_ = 1536 °C). Such different properties of copper can be explained in terms of the considerably lower value of “bonding energy” $\varepsilon_{\mathrm{Cu}-\mathrm{Cu}}$ in L–J potential than the iron counterpart $\varepsilon_{\mathrm{Fe}-\mathrm{Fe}}$ (both values are given in Figure 4). It is worth noticing that “bonding lengths” (or “lattice constants) $a_{ij}=\sqrt[{6}]{2}\sigma_{ij}$ are almost the same for both metals (the values of L–J potential $\sigma_{ij}$ parameters are also specified in Figure 4).

The experimental values of friction coefficients for pure metallic elements are hardly accessible in the literature owing to the fragility of the material as well as the strong dependence of polycrystallinity and surface state on the casting process. Moreover, the fundamental question of tribology is how to relate the atomic friction coefficients to the coefficients of macroscopic friction. This problem is far beyond the frame and the aim of the present work. The main goal is to make very qualitative comparisons between the simulation outcomes with experimental data concerning possibly similar metal combinations. The experimentally determined coefficient of dry sliding friction seems to be the most adequate for such a comparison, because, e.g., the static friction coefficient is predominantly dependent on macro- and microasperities of the surface. In the case of the cast iron–cast iron friction pair, the dry sliding friction value was estimated at about 0.15 [36]. For the case of the copper–copper pair, the dry sliding friction coefficient value was determined to be about 0.4 [47] after the occurrence of strong plastic deformations at the preliminary stage of the sliding process. Thus, as expected, the friction coefficient is higher for copper, which is a softer metal than iron. This coincides well with the presented simulation results of the atomic friction. Nevertheless, a thorough experimental study in [47] demonstrates that, for very flat and ideally clean Cu surfaces, the quasi-static friction coefficient value can rise even over 1.0 owing to the adhesion effect. In the case of Cu-Fe iron sliding, our simulations predict atomic friction coefficient values higher than for Fe-Fe pairs, but smaller than that for the Cu-Cu pair (as seen in Figure 4). This qualitative trend predicted within our simulations is in plain accordance with the experimental data [48] demonstrating a decrease of COF value from 0.4 for copper–copper pairs to 0.29 for the cast iron–copper tribopair.

The Cu-Fe case discussed above can be helpful in the description of the atomic sliding friction after placing the Cu nanoparticle between Fe atomic surfaces. Although the atomic friction coefficient of the Cu-Cu atomic pair is higher than that of the Fe-Fe pair, it does not actually lead to worsening of the effective tribological properties after adding Cu nanoparticles to the Fe-Fe tribopair. One can expect that, at the initial stage, the rolling of particles significantly reduces the friction force. At the same time, the particles of soft metal are being crushed and smashed. After such a process, a significant increase of the friction coefficient would be expected, but this is not the case because of the auto-repairing—or self-improving—of the friction surfaces [31]. At the microscopic level, it means the migration of “soft” metal atoms to the atomic vacancies at the surface. Consequently, the surface becomes more atomically flat, which results in reducing the sliding friction. On the other hand, the performed simulations predict a considerable increase of friction force and friction coefficient when significantly approaching the “slider” atom to the “slab” one. For the vertical distance *h* of smaller than half of the lattice constant, one can expect the atomic sliding friction coefficient to be higher than 1.0 (already not displayed in Figure 4)—i.e., even greater than for the macroscopic static friction. Such circumstances are possible in the case of surface roughness in the form of atomic vacancies and steps. That is why the self-improving process of the surface is so important. The copper atoms seem to be effective in filling asperities and creating flat areas of the surface owing to Cu inclusions. As shown in Figure 4, in the case of the Cu-Fe(Cu,Fe,Cu) pair, when the iron atom in the slab is surrounded by one Fe atom and two Cu atoms as inclusions, the atomic friction takes a value between those for the pure Fe-Fe(Fe,Fe,Fe) and Cu-Cu(Cu,Cu,Cu) pairs in the whole range of loading pressure. This tendency can be physically explained within the present model as a natural consequence of binary Lorentz–Berthelot mixing rules (2). The Cu-Fe(Fe,Fe,Fe) pair corresponds to the friction of Cu nanoparticle atom with Fe atoms of the slab without any inclusions. In this case, COF values are almost as low as for the pure Fe-Fe case. Thus, one can conclude that Cu nanoparticles are in general profitable additives, because at the atomic level, they elevate COF very slightly and, owing to the mechanical softness, they reveal surface auto-repairing features, which leads to the final reduction of the effective COF. Other simulations [31] predict a friction reduction when adding copper together with graphite.

**Figure 5:**
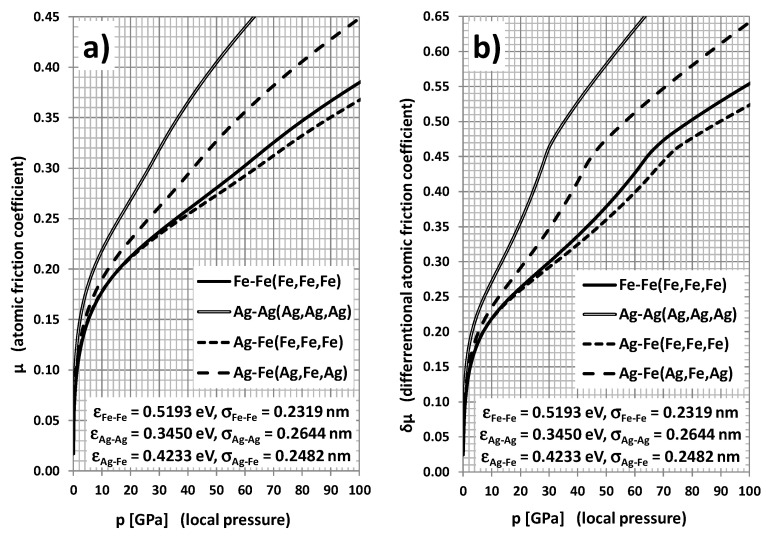
Regular (**a**) and differential (**b**) atomic friction coefficient dependence on local pressure for different atomic pairs: Fe-Fe(Fe,Fe,Fe), Ag-Ag(Ag,Ag,Ag), Ag-Fe(Fe,Fe,Fe), and Ag-Fe(Ag,Fe,Ag). Atoms specified in parentheses constitute a rigid environment of “elastic” atom of base.

The scope of the performed simulations also comprised the case of silver and molybdenum inclusions originating from the corresponding nanoparticles’ additives present in the initial phase of friction. In the case of the Ag-Ag atomic pair, the coefficient of friction is significantly higher than for the Fe-Fe pair and Cu-Cu pair (Figure 5), presumably owing to noticeably smaller “bonding energy” ε and longer “bonding lengths” $a_{ij}=\sqrt[{6}]{2}\sigma_{ij}$, which lead to very soft mechanical properties (*E* = 74 GPa, *G* = 28 GPa, *UTS* = 0.13 GPa) and a low melting point *T*_Ag_ = 962 °C. This coincides with the extraordinarily great experimental value of dry sliding friction of the order of 1.4—practically the same as for the static case [36]. As in the case of Cu, the friction coefficient for the Ag-Fe pair (without Ag inclusions in the “slab”) takes intermediate values; nevertheless, in the case with Ag inclusions, the coefficient values drop below those for pure Fe-Fe friction. This is a very promising result in terms of practical applications.

**Figure 6:**
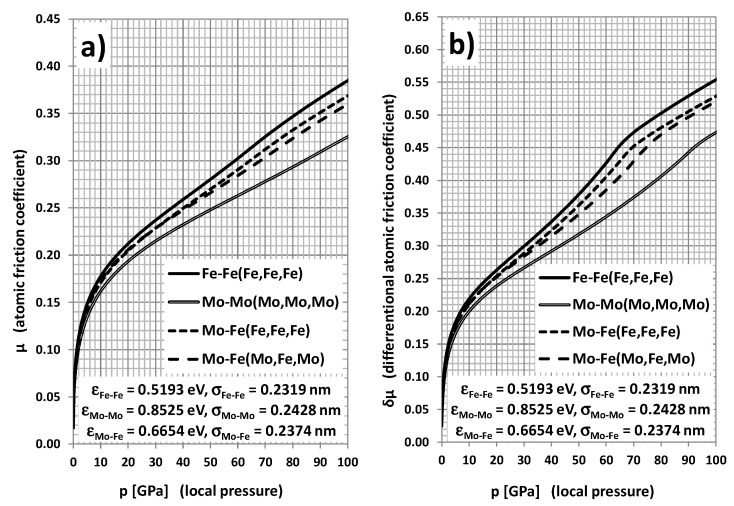
Regular (**a**) and differential (**b**) atomic friction coefficient dependence on local pressure for different atomic pairs: Fe-Fe(Fe,Fe,Fe), Mo-Mo(Mo,Mo,Mo), Mo-Fe(Fe,Fe,Fe), and Mo-Fe(Mo,Fe,Mo). Atoms specified in parentheses constitute a rigid environment of the “elastic” atom of base.

When considering the option of molybdenum additive, the obtained results of the simulations are very different from previous ones. The coefficient of Mo-Mo pair friction is significantly lower than for Fe-Fe (Figure 6). This is a consequence of hard mechanical properties (*E* = 329 GPa, *G* = 126 GPa, *UTS* = 0.67 GPa) and a high melting point *T*_Mo_ = 2620 °C owing to significantly higher “bonding energy” ε with comparable “bonding lengths” $\sqrt[{6}]{2}\sigma_{ij}$. The values of atomic friction coefficient for the Mo-Fe pair (both without and with Mo inclusions in the “slab”) are considerably lower than for Fe-Fe; however, the case of inclusions is especially beneficial. This lowering of atomic friction owing to Mo additives should have a positive impact on the macroscopic dry friction; however, the process of self-creating molybdenum inclusions can be impeded because of the hardness of this metal.

There is a lack of reliable information in the literature on the experimental values of the friction coefficient of pure molybdenum; however, a very recent paper [49] reports that amorphous Fe–Mo–Cr–Co coatings (with a dominating contribution of Mo) cause the reduction of COF by a factor of 3 compared with the 316L stainless steel. These results demonstrate a great applicational potential of metallic molybdenum in tribology and biotribology [1]. This is a novel perspective besides the molybdenum disulfide (MoS_2_). Both experimental and simulative works confirm that molybdenum disulfide in various forms (thin layered coatings, nanoparticles) reveals excellent lubricative properties [50,51,52,53,54] and reduces the COF value well below 0.1 for different kinds of tribopairs. The mechanism of this reduction is clear in terms of interlayer sliding owing to the weak van der Waals coupling of atomic layers in this material. However, MoS_2_ is a less stable phase than intermetallic associations with molybdenum.

## 4. Conclusions

In this paper, comparative studies on metallic inclusions role in atomic-level friction were performed using pseudo-static approximation. The simplicity and time efficiency of this model allow considering various atomic tribopairs with different atomic surroundings and a very wide range of loading forces. It has been demonstrated that, although Cu-Cu atomic friction is higher than Fe-Fe ones (by about 14%), the Cu inclusions in the Fe surface can reduce the COF via a self-repairing process. This is a crucial feature because Cu nanoparticles are a cheap and commonly used nano-lubricant for dry sliding friction and, as demonstrated in numerous MD simulations, copper easily fills the atomic vacancies at the rough friction surfaces. The COF of Cu atoms with Fe(Cu,Fe,Cu)-type inclusion is ca. 7% higher than for the Fe-Fe atomic pair and ca. 6% lower than the Cu-Cu pair. Moreover, it has been demonstrated that the friction of Cu atoms on a pure Fe slab is almost the same as for the Fe-Fe pair. In the case of Ag-Ag atomic friction, the COF value is significantly higher than for Fe-Fe (by ca. 47%); however, the performed simulations point to a significant drop in the COF in the case of Ag-Fe pairs—even below the value corresponding to the Fe-Fe one (by ca. 3%). This means that Ag-based nanoadditives, although more expensive than Cu ones, could be a good solution for some critical tribological systems. The results of the simulations obtained for the molybdenum convince one that Mo-Mo atomic-level friction is considerably lower than for the Fe-Fe pair (by ca. 13%) and in the case of Mo friction with Fe(Mo,Fe,Mo)-type inclusions (by ca. 5%). These facts point to the metallic Mo coatings as efficient reducers of dry friction, revealing potentially higher durability and stability than MoS_2_ layers [50,51,52,53,54]. Their applicational potential would also be enhanced by MO_3_ lamellar additives [55].

The main contribution of the present work was the development of the time-efficient method for pre-estimation of atomic COF and its application to specific tribopairs (including different atomic inclusions). The proposed fast method of simulation can also be applied to other atomic tribopairs. In general, it could be the first-step assessment procedure before the target large-scale MD simulations of systems characterized by various initial degrees of surface roughness supplemented by a variety of metallic additives. A natural next-step continuation of this research will be a consideration of local temperature increase, the influence of sliding velocity, and various geometries of surface roughness and the distribution of inclusions. The performed comparison between simulated atomic friction and the experimental data has only an illustrative value because the conditions differ in terms of length scale, nominal load, and other aspects. Nevertheless, atomic-level friction is one of the significant components of the real friction phenomenon. In the present work, the frictional base (substrate) was assumed to be flat both without inclusions and with heteroatomic inclusion cases. Within the future project, the consideration of atomic steps is prevised. In order to achieve better comparison compatibility, it would be advisable to schedule very systematic experiments on various atomic tribopairs using friction force microscopy.

## Data Availability

Not Applicable.
